# Rising burden of Hepatitis C Virus in hemodialysis patients

**DOI:** 10.1186/1743-422X-8-438

**Published:** 2011-09-13

**Authors:** Sanaullah Khan, Sobia Attaullah, Ijaz Ali, Sultan Ayaz, Naseemullah, Shahid Niaz Khan, Sami Siraj, Jabbar Khan

**Affiliations:** 1 Molecular Parasitology and Virology Laboratory, Department of Zoology, Kohat University of Science and Technology, Kohat 26000, Khyber Pakhtunkhwa, Pakistan; 2 Department of Zoology, Islamia College (Public Sector University) Peshawar, University Campus, 25120, Khyber Pakhtunkhwa, Pakistan; 3 Institute of Biotechnology and Genetic Engineering, KP University of Agriculture Peshawar, Khyber Pakhtunkhwa, Pakistan; 4 Lady Reading Hospital Peshawar, Khyber Pakhtunkhwa, Pakistan; 5 Institute of Basic Medical Sciences, Khyber Medical University, Peshawar, Pakistan; 6 Department of Biological Sciences, Gomal University, D.I. Khan, Pakistan

**Keywords:** Dialysis patients, HCV, HCV Genotype, Epidemiology, Pakistan

## Abstract

**Aim:**

High prevalence of Hepatitis C virus (HCV) has been reported among the dialysis patients throughout the world. No serious efforts were taken to investigate HCV in patients undergoing hemodialysis (HD) treatment who are at great increased risk to HCV. HCV genotypes are important in the study of epidemiology, pathogenesis and reaction to antiviral therapy. This study was performed to investigate the prevalence of active HCV infection, HCV genotypes and to assess risk factors associated with HCV genotype infection in HD patients of Khyber Pakhtunkhwa as well as comparing this prevalence data with past studies in Pakistan.

**Methods:**

Polymerase chain reaction was performed for HCV RNA detection and genotyping in 384 HD patients. The data obtained was compared with available past studies from Pakistan.

**Results:**

Anti HCV antibodies were observed in 112 (29.2%), of whom 90 (80.4%) were HCV RNA positive. In rest of the anti HCV negative patients, HCV RNA was detected in 16 (5.9%) patients. The dominant HCV genotypes in HCV infected HD patients were found to be 3a (n = 36), 3b (n = 20), 1a (n = 16), 2a (n = 10), 2b (n = 2), 1b (n = 4), 4a (n = 2), untypeable (n = 10) and mixed (n = 12) genotype.

**Conclusion:**

This study suggesting that i) the prevalence of HCV does not differentiate between past and present infection and continued to be elevated ii) HD patients may be a risk for HCV due to the involvement of multiple routes of infections especially poor blood screening of transfused blood and low standard of dialysis procedures in Pakistan and iii) need to apply infection control practice.

## Introduction

Hepatitis C virus (HCV) infection is a major public health problem, with an estimated global prevalence of 3% occurring in about 180 million carriers and approximately 4 million people have been newly infected annually [1]. The prevalence of HCV infection among dialysis patients is generally much higher than healthy blood donors [2] and general population [3]. Studies held in dialysis centers from different countries revealed that prevalence ranges form 1-84.6% [2,4] and there is a particular concern because HCV chronic infection causes significant morbidity and mortality among patients undergoing hemodialysis (HD) [4].

In Pakistan currently, approximately 10 million people are suffering from this tremendous disease which cover 6% of the overall population. A high prevalence of HCV Ab (38% weighted average) was described in the studies of patients undergoing chronic dialysis in Pakistan [5]. The spread of HCV in Pakistan is fuelled due to lack of education and awareness of disease, shortage of medically qualified and scientifically trained health care workers especially dentists, lack of health infrastructure such as use unsterilized instruments, use of high numbers of therapeutic injections and practice of daily face and armpit shaving in community barber shops [6,7]. New HCV infection was evidently more frequent at dialysis centers with higher anti-HCV prevalence and failure in infection control measures. In some countries, both prevalence and incidence remain very high, indicating major ongoing nosocomial transmission, probably due to the limited resources available to treat a rapidly growing HD population [8].

The striking genetic heterogeneity of the RNA genome of HCV is well recognized [9,10]. On the basis of molecular relatedness, HCV is classified into 11 major genotypes: 1 through 11, among which first six are major player of infection globally [9]. On the basis of phylogenetic analysis, over 80 subtypes and minor variants referred to as "quasispecies are existing [10], which differ by 20% to 23% on the basis of full length genomic sequence comparisons subtype [11]. Identification of HCV genotype does not influence disease presentation but is important for its predictive value in term of antiviral therapy, counseling and management [12]. Counseling is indeed a necessity in order to minimize the risk of transmission of HCV infection to others [13]. This study investigated the subtypes of HCV infection and correlate genotypes of the HD patients with the demographic data and risk factors. This study also evaluation the prevalence of HCV with the past studies in HD patients conducting in different regions of Pakistan.

## Materials and methods

### Study Sample and Data Collection

384 HD patients were randomly selected collected from three hospitals of Peshawar, Khyber Pakhtunkhwa: Khyber Teaching Hospital, National Diagnostic Dialysis Center and Dialysis Ward Hayatabad Medical Complex. All patients were briefed about the study and proper willing consent was signed.

All patients were interviewed for demographic data and risk factors to HCV infections including history of number of blood transfusion, intravenous drug use (IDU), surgical interventions, and dental treatment, multiple sexual partners, barber shop, piercing instruments and exposure to known HCV-positive persons, number of years on dialysis and change of the center. Blood (5CC in sterile syringes) were collected from HD patients; sera were separated in two aliquots and frozen at -70°C for HCV RNA detection and genotyping.

### Antibodies Screening

All the subjects were screened for anti HCV antibodies third generation test according to the manufacture instructions (Accurate Diagnostics USA).

### RNA Isolation, PCR amplification and Detection

RNA was isolated from 100 μL serum, using RNAzole RNA purification kit (Trizole Inc. USA) as per manufacturer protocols. HCV RNA was Reverse transcribed with 200 U of Maloney murine leukemia virus reverse transcriptase (Fermentas USA) and amplified with nested primers specific for 5' untranslated region of HCV genome. Amplified product was subjected in 2% agarose gel for electrophoresis.

### HCV genotyping

HCV RNA positive samples were genotyped with primers specific for core region of different HCV types by type specific PCR as mentioned elsewhere [14].

For contamination control RNA extraction, cDNA amplification and electrophoresis were carried in separate areas. Both negative and positive controls were run parallel to the patient samples in each batch.

### Statistical Analysis

χ^2 ^test were used to accesses the association between HCV positivity and variables. Statistical significance was evaluated at 0.05 levels.

## Results

This study involved 384 patients treated with HD and was screened for anti HCV and tested for HCV RNA and HCV genotypes during January 2010-April 2010. The mean age of the patients was 40.9 ± 5.54 years (age rang15-75 years). 244 were male (mean age 40.4 ± 8.4) and 140 were female (age range 41.7 ± 10.07), 340 patients were married (mean age 42.7 ± 8.03) and 48 were unmarried (mean age 28 ± 4). The mean duration of HD was 6.7 years (range 1-12 years). Change of dialysis centre was noted in twelve patients. Of the total, 112 (29.2%) patients were anti HCV positive; 48 were females (42.85%) and 64 were males (57.14%). HCV RNA was detected in 90 (80.4%) anti HCV positive patients and in 16 (5.9%) anti HCV negative patients. None positive patients had exposure to known HCV-positive persons.

### Genotype distribution

**Figure 1:**
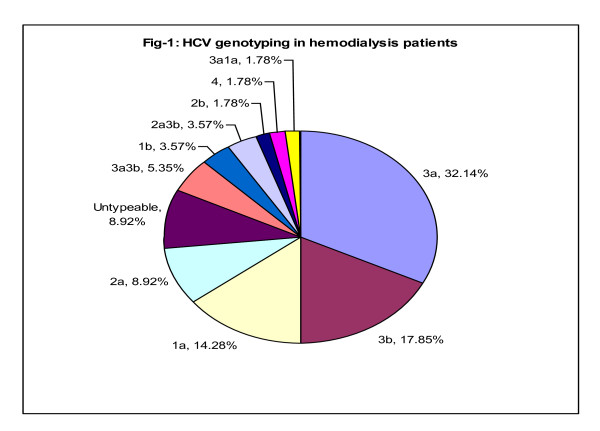
**HCV genotyping in hemodialysis patients**.

In current study the genotype distribution was as 3a (42.85%), 3b (23.21%). 2a (14.28%), 1a (16.07%), untypable (8.92%), 1b (3.57%) and 4 (1.78%) (Figure 1).

### Risk assessment for HCV infection

Table 1 showed the genotype distribution (%) according to age and gender. HD patients were classified into four age groups. The frequency distribution of genotypes in relation to age revealed that genotype 3a was most common in nearly all age groups while 3b and 2a were comparatively less frequent. Age group with > 46 years were more affected with mixed genotype while untypable genotype were more common in age group < 45 years. Genotype 3 (a and b) were common in female as compare to male while genotype 1a, untypable and mixed were more common in male as compared to female. Genotype 1b and 4a were found only in male. Prevalence of genotype subtypes within age groups (χ^2 ^= 22.076, p = 0.004) were found significant while gender (χ^2 ^= 42.48, p = 0.0113) and marital status (χ^2 ^= 62.803, p = 0.247) of the patients were not statistically significant.

**Table 1:** HCV genotype distribution (%) according to gender and age

Gender	Genotypes of HCV									
	
	1a	1b	2a	2b	3a	3b	4a	Untypable	Mixed	Total
**Male**	12	4	4	2	14	8	2	8	10	**64**

**Female**	4	0	6	0	22	12	0	2	2	**48**

**Age in years**										

**15-30**	2	2	2	0	6	4	2	4	0	**22**

**31-45**	4	0	2	0	8	6	0	4	0	**24**

**46-60**	6	0	0	0	12	6	0	2	4	**30**

**61-75**	4	2	6	2	10	4	0	0	8	**36**

**Total**	16	4	10	2	36	20	2	10	12	**112**

Table 2 showed the distribution of different HCV genotypes with relation to different risk factors in HD patients. Mixed genotype was more prevalent in HD patients with history of blood transfusion and barber shops. Mixed and untypable genotype was found significantly more often in HD patients with history of mean number of 2.28 blood transfusions and genotype 3a, 3b and mixed genotype were found more commonly in patients with mean number of 4.61 blood transfusions. IDUs were found with untypable and mixed genotypes only. In patients infected through barber shops, genotype 3a and 2a were more prevalent, followed by 3b and 1a genotypes. The most prevalent risk factor for untypable genotype was found to be dental treatment and barber shop.

**Table 2:** Distribution of different HCV genotypes with relation to different risk factors in HD patients

Variables	Distribution of different genotypes of HCV in HD patients									
	
	1a	1b	2a	2b	3a	3b	4a	untypable	mixed	Total
**H/O of blood transfusion**	14	2	7	1	36	20	1	6	12	99

**H/O of surgery**	0	0	2	0	2	1	0	0	1	6

**H/O of IDU**	0	0	0	0	0	0	0	1	3	4

**H/O of multiple sexual exposure**	0	1	0	0	1	0	0	0	1	3

**House hold contacts**	0	0	0	0	0	0	0	0	0	0

**H/O of dental treatment**	1	2	1	0	5	2	0	7	1	19

**H/O of barber shop**	12	3	9	2	34	16	2	10	12	100

**H/O of needle sharing**	0	0	0	0	1	0	0	0	1	2

**H/O piercing procedures**	1	2	1	0	8	4	0	2	2	20

Regarding the duration on HD, 42% patients were being dialyzed during 1997-2009 and 58% from 2004-2009 with most prevalent genotypes 3a, 3b and 2a, and untypable and mixed genotypes, respectively.

## Discussion

The HCV infection continues to be a major disease burden on the world. For example, the prevalence of HCV antibodies among dialysis patients has been reported to range from: 8 to 36% in North America, 39% in South America, 1 to 54% in Europe, 17 to 51% in Asia1.2 to 10% in New Zealand and Australia [15]. The first description of HCV in Pakistan was recorded in 1992 and about 6%, i.e. at least ten million persons are carriers among a population of 140 million, showed there is no proper review of HCV and it is becoming a Herculean challenge. With the current disease burden, Pakistan has left behind the surrounding countries like India, Nepal, Myanmar, Iran and Afghanistan [1]. HCV gained importance particularly as major complication in multiple transfused patients during the last decades especially in the countries where HCV is more prevalent in general population and amongst the blood donors [16]. This was the first study conducted in patients treated with HD in Pakistan to determine the distribution of HCV genotypes and their interrelation with risk factors. Even though considerable progress has been attained during the last years, HCV prevalence rate among HD patients does not seem to have changed considerably [17].

The incidence and prevalence of HCV among dialysis patients varies markedly from country to country [18-20] and among dialysis centers within a single country [19]. HCV prevalence is much higher in developing countries as compared to developed world [21]. Table 3 showed that first paper in Pakistan in this line of literature was published in 1999 and till yet six different reports consisting of small sample size (ranging from 28-190) showed that an HCV percent prevalence of 26.02% among the HD population, rate of Prevalence become high in past as compared to recent studies. The prevalence rate of HCV in this study from Khyber Pakhtunkhwa stated that HCV infection was persistent public health concern in dialysis patients. This study comprised high prevalence rate might be due to large sample size as compared to rest of all.

**Table 3:** HCV prevalence in HD patients in different areas of Pakistan

Reference	Place	Study year	Publication year	Method	Sample size	HCV positive (%)
Gul and Iqbal [21]	Sheikh Zayed Postgraduate Medical Institute, Lahore	Dec1999	2003	ELISA	50	33(68)

Shafiq *et al *[22]	Ganga Ram Hospital, Lahore	2001-2002	2002	ELISA	190	47(24.7)

Shall *et al *[23]	Sheikh Zayed hospital, Lahore	2000-2002	2003	ELISA	122	24(19.7)

Khokhar *et al *[24]	Shifa International Hospital, Islamabad	2002-2003	2005	ELISA	97	23(23.7)

Zarkoon *et al *[25]	Sandeman Provincial Hospital, Quetta	Jan 2006-June 2007	2008	ELISA	97	23(23.7)

Ali *et al *[26]	Khyber Pakhtunkhwa	-	2011	PCR	28	2(07)

Current study	KTH, HMC & National Diagnostic Dialysis Center, Peshawar	Jan-April2010	2011	ELISA	384	29

A panel of 30 top gastroenterologists of the country met in 2004 at a conference and reported that 75%-90% of HCV Pakistani patients were harboring genotype 3a, followed by genotype 1 [9], also confirmed in current and in other different Pakistani population [1,7,27,28].

The prevalence was higher among the males. High prevalence in male as compared to female could be due to their exposure to various HCV risk factors [6] particularly barber community and multiple sexual exposures [10]. In current study 3 was more prevalent in females and 1 in male, as stated in a study that genotype 1 was more common in male as compared to females [27]. For HCV prevalence and genotype distribution statistically no significant difference was found between male (57.14%) and female patients, verified by other study [2,12,17]. Old age groups were found more infected with HCV. It has been suspected that fragile health structure, unsterilized instruments and use of contaminated razor by barbers may be contributing to the spread of HCV [5,7].

Blood transfusion(s) constitute a part of treatment in many HD patients and thus exposed greatly to HCV [2,14,29]. The risk of hepatitis transmission through blood transfusion is considered to be high in Pakistani population due to a lack of appropriate screening of blood in past. Several studies confirmed the prevalence of HCV among people with a history of blood transfusion before the advent of blood screening procedures in Pakistan [6,9]. Percentage prevalence of HCV was 4.95% ± 0.53% in the general adult population and 7.94 ± 1.49% in multi transfused population [9]. The prevalence found in this study (75%) was greater than that estimated for general adult population in the country. The multitransfused patients in this study was more prone to HCV, also supported by various studies [2,14,20,29,30] but could not recognizes as independent risk factor in other studies [13,17,20,30].

Needle sharing and household contacts were not significant risk factors in this study, similar to other literature [3,15]. There is an indication that environment of dialysis treatment itself function as a vehicle in dissemination of HCV among HD patients [29]. A high prevalence of patients with HCV infection in HD facilities has been considered a risk factor for transmission of the infection [8]. Several reports have linked a high incidence of HCV infection in dialysis patients who shared dialysis machines in dialysis unit [15].

This study showed a statistically significant difference in the prevalence of HCV infection between patients who were on dialysis for more than five years, and patients who were on dialysis for less than five years. Various other studies [2,13,15,18,29-31] confirmed that duration of HD was considered one of the risk factors for acquiring HCV infection. Thus strict application of infection control precautions including early screening of patients for anti-HCV and separate machines for anti-HCV positive patients led to a decline in the incidence of seroconversion in HD unit [15].

## Conclusion

It is concluded from pervious and current study that HCV prevalence in HD patients continued to be elevated in Pakistan. HCV genotype 3 accounts for approximately one-thirds of HCV-infected HD patients and the distribution of HCV genotypes in HD patients is similar to that in other population of Pakistan. High prevalence of HCV in HD patients demonstrated that environment of dialysis treatment itself function as a vehicle in dissemination of HCV among HD patients as well as they are exposed to the same community risk factors as general population.

## Competing interests

The authors declare that they have no competing interests.

## Authors' contributions

SK, SA and IA were involved in the designing the study. NU helped in collection of samples/data. SA and SNK did experimental work. SS and JK revised critically the manuscript. All the authors read and approved the final manuscript.
